# Mechanism of inhibitory effect of atorvastatin on resistin expression induced by tumor necrosis factor-α in macrophages

**DOI:** 10.1186/1423-0127-16-50

**Published:** 2009-05-27

**Authors:** Kou-Gi Shyu, Su-Kiat Chua, Bao-Wai Wang, Peiliang Kuan

**Affiliations:** 1 Division of Cardiology, Shin Kong Wu Ho-Su Memorial Hospital, Taipei, Taiwan; 2 Graduate Institute of Clinical Medicine, College of Medicine, Taipei Medical University, Taipei, Taiwan

## Abstract

Atorvastatin has been shown to reduce resistin expression in macrophages after pro-inflammatory stimulation. However, the mechanism of reducing resistin expression by atorvastatin is not known. Therefore, we sought to investigate the molecular mechanisms of atorvastatin for reducing resistin expression after proinflammatory cytokine, tumor necrosis factor-α (TNF-α) stimulation in cultured macrophages. Cultured macrophages were obtained from human peripheral blood mononuclear cells. TNF-α stimulation increased resistin protein and mRNA expression and atorvastatin inhibited the induction of resistin by TNF-α. Addition of mevalonate induced resistin protein expression similar to TNF-α stimulation. However, atorvastatin did not have effect on resistin protein expression induced by mevalonate. SP600125 and JNK small interfering RNA (siRNA) completely attenuated the resistin protein expression induced by TNF-α and mevalonate. TNF-α induced phosphorylation of Rac, while atorvastatin and Rac-1 inhibitor inhibited the phosphorylation of Rac induced by TNF-α. The gel shift and promoter activity assay showed that TNF-α increased AP-1-binding activity and resistin promoter activity, while SP600125 and atorvastatin inhibited the AP-1-binding activity and resistin promoter activity induced by TNF-α. Recombinant resistin and TNF-α significantly reduced glucose uptake in cultured macrophages, while atorvastatin reversed the reduced glucose uptake by TNF-α. In conclusion, JNK and Rac pathway mediates the inhibitory effect of atorvastatin on resistin expression induced by TNF-α.

## Background

Resistin is an adipocyte-secreted molecule induced during adipocyte differentiation. Recombinant resistin up-regulates cytokines and adhesion molecule expression on human endothelial cells [1,2], suggesting a potential role in atherosclerosis. Resistin has been shown to have potent proinflammatory properties [3]. Resistin promotes endothelial cell activation and causes endothelial dysfunction of porcine coronary arteries [4]. Recently, resistin was found to have a potential role in atherosclerosis because resistin increases MCP-1 and sVCAM-1 expression in vascular endothelial cells and resistin promotes vascular smooth muscle cell proliferation [5,6]. More recently, resistin was found to be secreted from macrophages in atheromas and promotes atherosclerosis [7]. Plasma resistin levels are correlated with markers of inflammation and are predictive of coronary atherosclerosis in humans, independent of plasma C – reactive protein. Resistin may represent a novel link between metabolic signals, inflammation, and atherosclerosis [8].

The 3-hydroxy 3-methyl glutaryl-CoA reductase (HMG-CoA reductase) inhibitors or statins have been proved to reduce inflammation and exert beneficial effects in the prevention of atherosclerosis progression [9]. The pleiotropic effect of statins, independent of their lipid-lowering effects have been described to improve endothelial function, stabilize atheroslerotic plaque, inhibit vascular smooth muscle cell proliferation as well as platelet aggregation, and reduce vascular inflammation [9]. Ichida et al reported that atorvastatin decreases resistin expression in adipocytes and monocytes/macrophages [10]. Atorvastatin decreased resistin mRNA expression in a dose- and time-dependent manner. However, the mechanism of reducing resistin expression by atorvastatin is not known. Therefore, we sought to investigate the molecular mechanisms of atorvastatin for reducing resistin expression after proinflammatory cytokine, TNF-α stimulation in macrophages.

## Materials and methods

### Drugs

Atorvastatin, a calcium salt of a pentasubstituted pryole, was supplied by Pfizer. A 10-mmole/l stock solution was made in 100% DMSO. Recombinant TNF-α protein and mevalonate were purchased from Sigma; Polyclonal Rac, and polyclonal phospho-Rac1 (Ser71) antibodies from Cell Signaling; Resistin antibody from R&D Systems; Rac 1 inhibitor, PD 98059, SB 203580, and anisomycin from CALBIOCHEM; Resistin siRNA from Invitrogen.

### Cell culture

Human peripheral mononuclear cells (PBMCs) were isolated from heparinized whole blood obtained from normal healthy volunteers by Ficoll-Hypaque gradient centrifugation. The cells were washed three times with sterile PBS and resuspended in RPMI 1640 supplemented with 10% fetal calf serum, 2 mmol/l L-glutamate and 1% penicillin/streptomycin. Monocytes were purified from PBMCs by positive selection using anti-CD14 magnetic beads according to the manufacturer's instructions. The cells were cultured for 4 days in RPMI 1640 supplemented with 10% fetal calf serum, 2 mmol/l L-glutamate and 1% penicillin/streptomycin. For experimental use, purified monocytes/macrophages were changed to serum-free RPMI-1640 supplemented with 2 mmol/l L-glutamate and 1% penicillin/streptomycin for 6 h, then treated with either 1 or 10 μmol/l of atorvastatin for 24 and 48 h.

### Western blot analysis

Cells were homogenized in modified RIPA buffer. Equal amounts of protein (15 μg) were loaded into a 12.5% SDS-polyacrylamide minigel, followed by electrophoresis. Protein samples were mixed with sample buffer, boiled for 10 min, separated by SDS-PAGE under denaturing conditions, and electroblotted to nitrocellulose membranes. The blots were incubated overnight in Tris-buffered saline (TBS) containing 5% milk to block nonspecific binding of the antibody. Proteins of interest were revealed with specific antibodies as indicated (1:1000 dilution) for 1 hour at room temperature followed by incubation with a 1:5000 dilution of horseradish peroxidase-conjugated polyclonal anti-rabbit antibody for 1 h at room temperature. Signals were visualized by chemiluminenescent detection. Equal protein loading of the samples was further verified by staining monoclonal antibody GAPDH. All Western blots were quantified using densitometry.

### RNA isolation and reverse transcription

Total RNA was isolated from cultured macrophages using the single-step acid guanidinium thiocyanate/phenol/chloroform extraction method. Total RNA (1μg) was incubated with 200U of Moloney-Murine Leukemia Virus reverse transcriptase in a buffer containing a final concentration of 50 mmol/L TrisCl (pH 8.3), 75 mmol/L KCl, 3 mmol/MgCl_2_, 20 U of RNase inhibitor, 1 μmol/L polydT oligomer, and 0.5 mmol/L of each dNTP in a final volume of 20 μL. The reaction mixture was incubated at 42°C for 1 h and then at 94°C for 5 min to inactivate the enzyme. A total of 80 μL of diethyl pyrocarbonate treated water was added to the reaction mixture before storage at -70°C.

### Real-time PCR

A Lightcycler (Roche Diagnostics, Mannheim, Germany) was used for real-time PCR. cDNA was diluted with nuclease-free water. 2 μL of the solution was used for the Lightcycler SYBR-Green mastermix (Roche Diagnostics): 0.5 μmol/L primer, 5 mmol/L magnesium chloride, and 2 μL Master SYBR-Green in nuclease-free water in a final volume of 20 μL. The primer used for mouse resistin was: forward, 5'-d(GTACCCACGGGATGAAGAACC)-3'; reverse, 5'-d(GCAGACCCACAGGAGCAG)-3'. The primer used for human resistin was: forward, 5'-d(TAAGCAGCATTGGCCTGG)-3'; reverse, 5'-d(CTGTGGCTCGTGGGATGT)-3'. GAPDH: forward, 5'-d(CATCACCATCTTCCAGGAGC)-3'; reverse, 5'-d(GGATGATGTTCTGGGCTGCC)-3'. The initial denaturation phase was 10 min at 95°C followed by an amplification phase as detailed below: denaturation at 95°C for 10 sec; annealing at 55°C for 5 sec; elongation at 72°C for 15 sec and for 30 cycles. Amplification, fluorescence detection, and post-processing calculation were performed using the Lightcycler apparatus. Individual PCR products were analyzed for DNA sequence to confirm the purity of the product.

### Electrophoretic mobility shift assay (EMSA)

Nuclear protein concentrations from macrophages were determined by Biorad protein assay. Consensus and control oligonucleotides (Santa Cruz Biotechnology Inc.) were labeled by polynucleotides kinase incorporation of [γ^32^P]-ATP. Oligonucleotides sequences included the activating protein 1 (AP-1) consensus 5'-CGCTTGATGACTCAGCCGGAA-3'. The AP-1 mutant oligonucleotides sequences were 5'-CGCTTGATGACTTGGCCGGAA-3'. After the oligonucleotide was radiolabeled, the nuclear extracts (4 μg of protein in 2 μl of nuclear extract) were mixed with 20 pmol of the appropriate [γ^32^P]-ATP-labeled consensus or mutant oligonucleotide in a total volume of 20 μl for 30 min at room temperature. The samples were then resolved on a 4% polyacrylamide gel. Gels were dried and imaged by autoradiography. Controls were performed in each case with mutant oligonucleotides or cold oligonucleotides to compete with labeled sequences.

### Promoter activity assay

A-741 to +22 bp rat resistin promoter construct was generated as follows. Rat genomic DNA was amplified with forward primer (ACGCGTCTCAGCGGTAGAGCTCTTG) and reverse primer (AGATCTGGAGAAATGAAAGGTTCTTCATC). The amplified product was digested with MluI and BglII restriction enzymes and ligated into pGL3-basic luciferase plasmid vector (Promega Corp. Madison, Wisconsin, USA) digested with the same enzymes. The resistin promoter contains AP-1 conserved sites (CT) at -51 to -52 bp. For the mutant, the AP-1 binding sites were mutated using the mutagenesis kit (Stratagene, La Jolla, California, USA). Site-specific mutations were confirmed by DNA sequencing. Plasmids were transfected into macrophages using a low pressure-accelerated gene gun (Bioware Technologies, Taipei, Taiwan) essentially following the protocol from the manufacturer. Test plasmid at 2 μg and control plasmid (pGL4-Renilla luciferases) 0.02 μg were cotransfected with gene gun in each well, and then replaced by normal culture medium. Following 4 hours of TNF-α stimulation, cell extracts were prepared using Dual-Luciferase Reporter Assay System (Promega) and measured for dual luciferase activity by luminometer (Turner Designs).

### Measurement of resistin concentration

Conditioned media from cultured macrophages under TNF-α stimulation and those from control cells were collected for resistin measurement. The level of resistin was measured by a quantitative sandwich enzyme immunoassay technique (R&D Systems). The lower limit of detection of resistin was 5 pg/mL. Both the intra-observer and inter-observer coefficient of variance were < 10%.

### Glucose uptake in macrophages

Macrophages were seeded on ViewPlate for 60 min (Packard Instrument Co., Meriden, Connecticut, USA) at a cell density of 5 × 10^3 ^cells/well in serum free medium with transferring 5 μg/mL, insulin 5. μg/mL for overnight. Recombinant TNF-α protein or conditioned medium were added to the medium. Glucose uptake was performed by adding 0.1 mmol/L 2-deoxy-D-glucose and 3,33 nCi/mL 2- [1,2-^3^H]-deoxy-D-glucose for various periods of time. Cells were washed with PBS twice. Non-specific uptake was performed in the presence of 10 μM cytochalasin B and subtracted from all of the measured value. MicroScint-20 50 μl was added and the plate was read with TopCount (Packard Instrument Co.). The radioactivity was counted and normalized to protein amount measured with a protein assay kit.

### Statistical analysis

The data were expressed as mean+S.E.M. Statistical significance was performed with analysis of variance followed by Dunnett's test for experiments consisting of more than two groups and with Student's t test for stretch at 10% and 20%. A value of P < 0.05 was considered to denote statistical significance.

## Results

### TNF-α increases resistin expression in human macrophages

**Figure 1:**
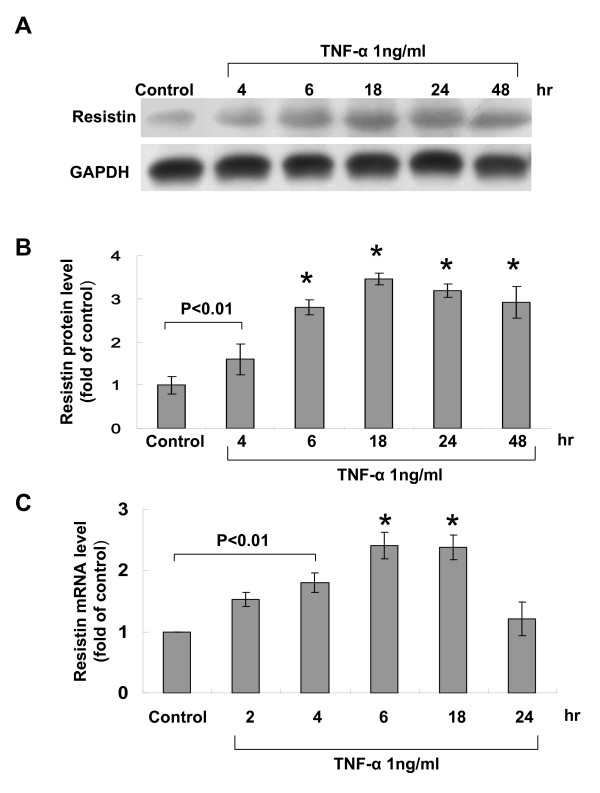
**Effect of pro-inflammatory cytokine, tumor necrosis factor-α (TNF-α) on resistin expression in cultured macrophages**. (A) Representative Western blots for resistin in macrophages treated with TNF-α for different periods of time. (B and D) (B) Quantitative analysis of resistin protein levels. The values from treated macrophages have been normalized to values in control cells. (n = 4 per group). *P < 0.01 vs. control. (C) Quantitative analysis of resistin mRNA levels. The mRNA level was measured by real-time PCR. The values from treated macrophages have been normalized to matched actin measurement and then expressed as a ratio of normalized values to mRNA in control cells (n = 3 per group). *P < 0.01 vs. control.

 Western blot was used first to investigate the effect of TNF-α on resistin expression in human macrophages. As shown in Fig. 1, resistin protein expression was significantly induced at 4 h after TNF-α (1 ng/mL) stimulation, maximally induced at 18 h, and remained elevated for 48 h. The expression of resistin messenger RNA was significantly induced by TNF-α from 4 to 18 h of stimulation (Fig. 1C).

### Atorvastatin inhibited the effect of TNF-α on resistin expression

**Figure 2:**
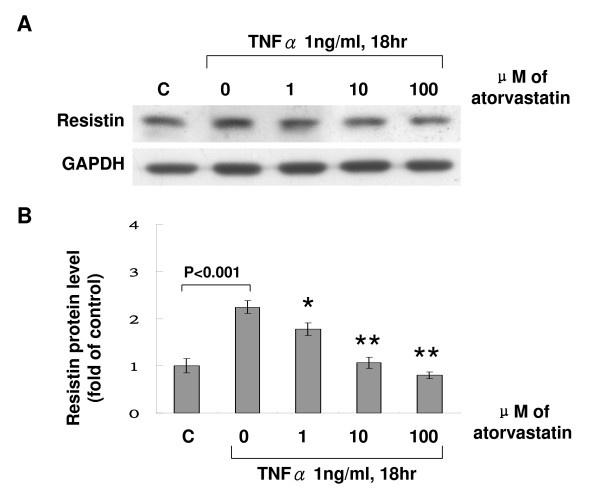
**Atorvastatin inhibits the resistin protein expression induced by TNF-α in a dose-dependent manner**. A, Representative Western blots for resistin in macrophages treated with TNF-α with or without atorvastatin. B, Quantitative analysis of resistin protein levels. The values from treated macrophages have been normalized to values in control cells. (n = 4 per group). *P < 0.05 vs. TNF-α. **P < 0.001 vs. TNF-α.

**Figure 3:**
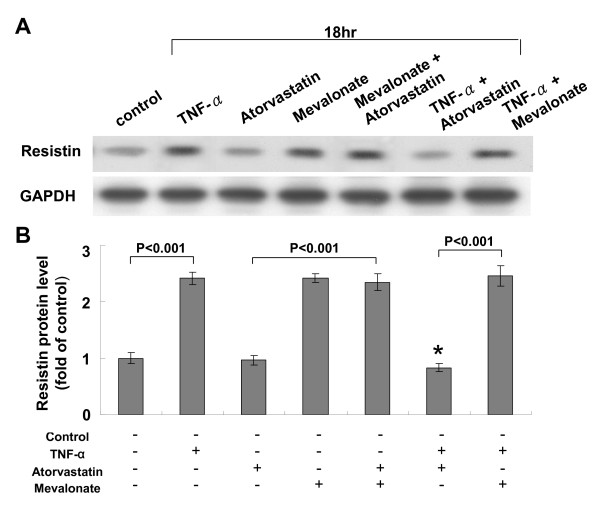
**Atorvastatin inhibits the TNF-α-induced resistin expression not via HMG-CoA reductase pathway**. A. Representative Western blots for resistin in macrophages treated with TNF-α, mevalonate with or without atorvastatin. B, Quantitative analysis of resistin protein levels. The values from treated macrophages have been normalized to values in control cells. (n = 4 per group). *P < 0.001 vs. TNF-α.

**Figure 4:**
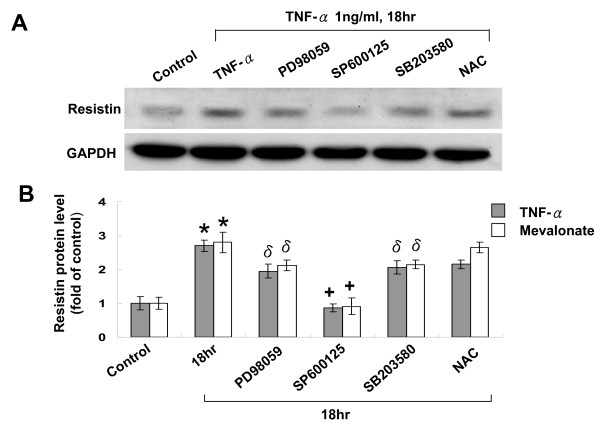
**JNK pathway is the main signal pathway mediating the induction of resistin protein expression by TNF-α and mevalonate**. A. Representative Western blots for resistin in macrophages treated with TNF-α with or without different inhibitors. B, Quantitative analysis of resistin protein levels. The values from treated macrophages have been normalized to values in control cells. (n = 4 per group).

To test whether atorvastatin can inhibit the effect of TNF-α on resistin expression in human macrophages, different doses of atorvastatin was added before TNF-α stimulation. As shown in Fig. 2, atorvastatin inhibited the resistin protein expression induced by TNF-α in a dose-dependent manner. Addition of mevalonate at 200 μM for 18 h induced resistin protein expression similar to TNF-α stimulation (Fig. 3A and 3B). However, atorvastatin did not have effect on resistin protein expression induced by mevalonate. Atorvastatin alone had neutral effect on resistin expression similar to control cells. Exogenous mevalonate did not reverse the resistin protein expression induced by TNF-α stimulation. This finding implicated that mevalonate has proinflammatory effect on resistin expression in human macrophages and atorvastatin inhibited the TNF-α-induced resistin expression not via HMG-CoA reductase pathway. SP600125, a potent inhibitor of JNK, completely attenuated the resistin protein expression induced by TNF-α and mevalonate (Fig. 4A and 4B). PD98059, a potent inhibitor of p42/p44 MAP kinase, and SB203580, a potent inhibitor of p38 MAP kinase, partially attenuated the resistin protein expression induced by TNF-α and mevalonate. NAC, an antioxidant scavenger, did not affect the resistin protein expression induced by TNF-α and mevalonate. TNF-α stimulation increased phosphorylation of JNK (Additional file 1), while TNF-α stimulation did not increase phosphorylation of p38 kinase and increased phosphorylation of ERK only after stimulation for 2 h. SP 60025 and Rac1 inhibitor significantly attenuated the phosphorylation of JNK induced by TNF-α stimulation. Atorvastatin also significantly reduced the phosphorylated JNK induced by TNF-α stimulation. These findings indicate that JNK pathway is the main signal pathway mediating the induction of resistin protein expression by TNF-α. Our data also demonstrated that Rac was also involved in TNF-α induced JNK activation.

### Rac pathway mediates the inhibitory effect of atorvastatin on resistin expression induced by TNF-α

**Figure 5:**
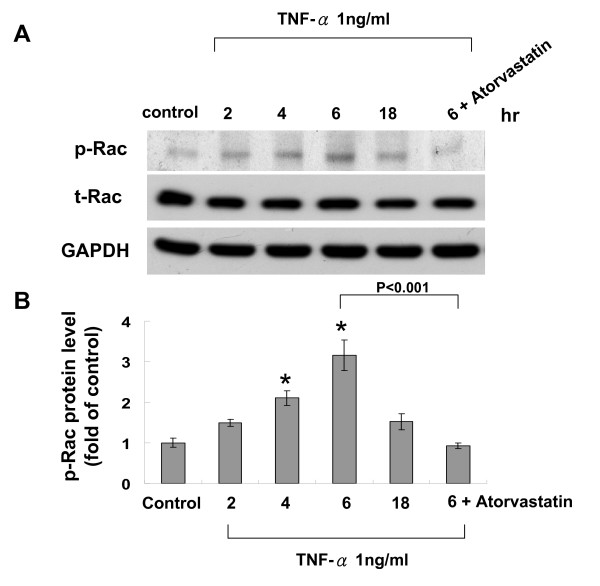
**TNF-α induces phosphorylation of rac protein expression in cultured macrophages**. A. Representative Western blots for phospho-rac and total rac in macrophages treated with TNF-α with or without atorvastatin. B, Quantitative analysis of phospho-rac protein levels. The values from treated macrophages have been normalized to values in control cells. (n = 4 per group). *P < 0.001 vs. control.

**Figure 6:**
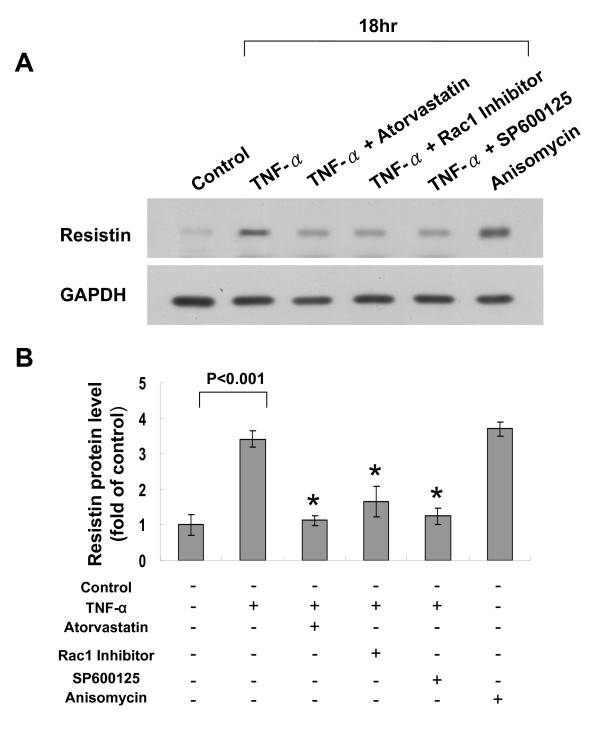
**Rac pathway mediates the inhibitory effect of atorvastatin on resistin expression induced by TNF-α**. A. Representative Western blots for resistin in macrophages treated with TNF-α with or without atorvastatin and different inhibitors. B, Quantitative analysis of resistin protein levels. The values from treated macrophages have been normalized to values in control cells. (n = 3 per group). *P < 0.001 vs.*P < 0.01 vs. TNF-α.

To investigate the atorvastatin inhibitory mechanism on induction of resistin by TNF-α, rac pathway was studied. As shown in Fig. 5, TNF-α induced phosphorylation of Rac in a dose-dependent manner. TNF-α did not have effect on total Rac. Addition of atorvastatin inhibited the phosphorylation of Rac induced by TNF-α. Rac-1 inhibitor almost completely attenuated the effect of TNF-α on resistin induction (Fig. 6). Anisomycin, an agonist of Rac, significantly increased the resistin protein expression similar to TNF-α. Rac1 inhibitor attenuated the induction of resistin protein expression by TNF-α, while rac1 inhibitor did not alter the resistin protein expression induced by anisomycin. As shown in Additional file 2, addition of mevalonate did not induce phosphorylation of Rac and total rac protein expression.

### TNF-α increases AP-1-binding activity and resistin promoter activity

**Figure 7:**
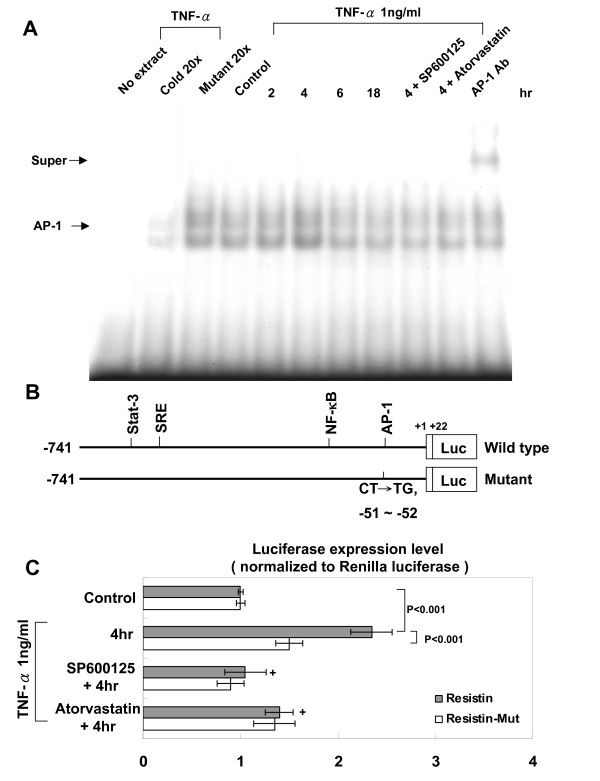
**TNF-α increases AP-1-binding activity and resistin promoter activity**. A. Representative EMSA showing protein binding to the AP-1 oligonucleotide in nuclear extracts of macrophages after TNF-α treatment in the presence or absence of atorvastatin or JNK inhibitor. Similar results were observed in another two independent experiments. A significant supershifted complex (super) after incubation with AP-1 antibody was observed. Cold oligo means unlabeled AP-1 oligonucleotides. B. Constructs of resistin promoter gene. Positive +1 demonstrates the initiation site for the resistin transcription. Mutant resistin promoter indicates mutation of AP-1 binding sites in the resistin promoter as indicated. C. Quantitative analysis of resistin promoter activity. Cultured macrophages were transiently transfected with pResistin-Luc by gene gun. The luciferase activity in cell lysates was measured and was normalized with renilla activity (n = 3 per group). *P < 0.001 vs. control. ^+^P < 0.01 vs. 4 h.

The EMSA assay showed that TNF-α increased AP-1 DNA-protein binding activity (Fig. 7). An excess of unlabeled AP1 oligonucleotide competed with the probe for binding AP1 protein, whereas an oligonucleotide containing a 2-bp substitution in the AP1 binding site did not compete for binding. Addition of SP600125 and atorvastatin 30 min before TNF-α stimulation abolished the DNA-protein binding activity induced by TNF-α. DNA-binding complexes induced by TNF-α could be supershifted by a monoclonal AP-1 antibody, indicating the presence of this protein in these complexes.

To study whether the resistin expression induced by TNF-α is regulated at the transcriptional level, we cloned the promoter region of rat resistin (-741~+22), and constructed a luciferase reporter plasmid (pGL3-Luc). The resistin promoter construct contains Stat-3, SRE, NF-κB, and AP1 binding sites. As shown in Fig. 7B and 7C, transient transfection experiment in macrophages using this reporter gene revealed that TNF-α stimulation for 4 h significantly caused resistin promoter activation. This result indicates that resistin expression is induced at transcriptional level by TNF-α. When the AP1 binding sites were mutated, the increased promoter activity induced by TNF-α was abolished. Moreover, addition of SP600125 and atorvastatin caused an inhibition of transcription. These results suggested that AP1 binding site in the resistin promoter is essential for the transcriptional regulation by TNF-α and that TNF-α regulates resistin promoter via JNK pathways.

### TNF-α stimulates secretion of resistin from macrophages and reduces glucose uptake

**Figure 8:**
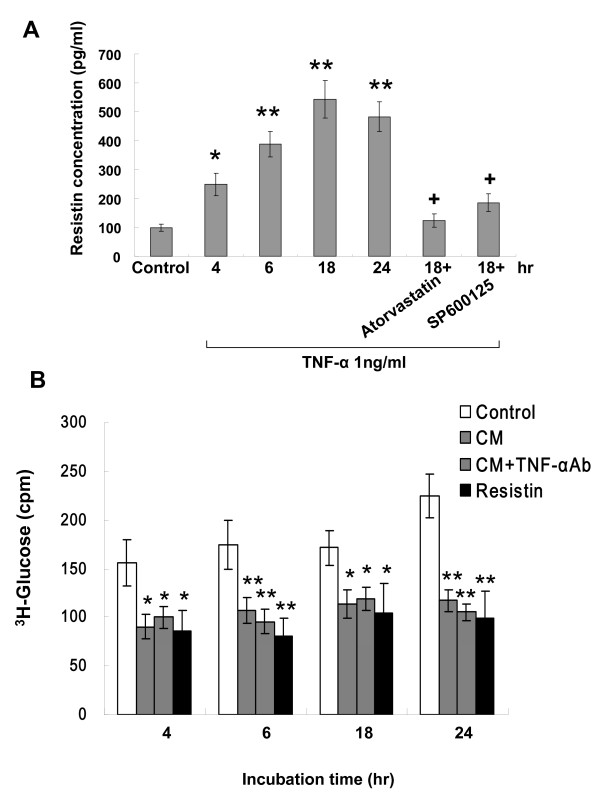
**TNF-α stimulates secretion of resistin from macrophages and reduces glucose uptake**. (A) TNF-α at 1 ng/mL significantly increasesd release of resistin from cultured macropghes at different stimulation periods. SP600125 and atorvastatin attenuated the effect induced by TNF-α (n = 3). *P < 0.01 vs. control. **P < 0.001 vs. control.^+^P < 0.001 vs. 18 hr. (B) Resistin and conditioned medium (CM) after TNF-α treatment had similar effect on reducing glucose uptake. Glucose uptake was measured in macrophages treated for 90 min with 20 μg/mL recombinant resisitn protein or conditioned medium from cultured macrophages after 1 ng/mL TNF-α stimulation with or without TNF-α antibody (TNF-α Ab). *P < 0.05 vs. control. **P < 0.01 vs. control. Data are from 3 independent experiments.

As shown in Fig. 8A, TNF-α significantly increased the resistin secretion from cultured macrophages from 4 to 24 h. The mean concentration of resistin rose from 98 ± 13 pg/mL before TNF-α stimulation to 542 ± 64 pg/mL after TNF-α stimulation for 18 h (P < 0.01). Pretreatment with atorvastatin or SP600125 significantly attenuated the secretion of resistin induced by TNF-α

Recombinant TNF-α protein at 1 ng/mL significantly reduced glucose uptake at various periods of incubation as compared to control macrophages without treatment (Additional file 3). As shown in Figure 8B, exogenous addition of conditioned medium from TNF-α stimulated macrophages and resisitn also increased glucose uptake in cultured macrophages. To eliminate the TNF-α effect on glucose uptake, anti-rat TNF-α antibody (5 μg/ml, purchased form R&D Systems) was added to the medium 1 hour before 2- [1,2-^3^H]-deoxy-D-glucose was added. The effect of cultured medium obtained from macrophages after TNF-α antibody treatment on reducing glucose uptake was similar to that of resistin. Addition of resistin siRNA or atorvastatin before recombinant resistin treatment reversed the glucose uptake to baseline levels (Additional file 3). This data indicates that resistin secreted from macrophage after TNF-α stimulation is functional.

## Discussion

Atherosclerosis has been considered an inflammatory disease. Inflammatory mediators such as TNF-α, interleukin-1 and C-reactiv protein paly an important role in atheogenesis. Resistin could stimulate expression of TNF-α, interleukin-1, 6 and 12 in cultured macrophages [3,11]. We have previously demonstrated a remarkable induction of resistin protein level even after stimulation with low level of TNF-α in vascular smooth muscle cells [12]. In this study, we further demonstrated that resistin protein and mRNA levels can be induced by TNF-α in cultured human macrophages. Macrophages and vascular smooth muscle cells are important components in the atheroma. These findings indicate that resistin is a promising target for controlling atherosclerotic disease.

Biomarkers that integrate metabolic and inflammatory signals are attractive candidates for defining risk of atherosclerotic cardiovascular disease [13]. Hyperresistinemia impairs glucose tolerance and induces hepatic insulin resistance in rodents [14], whereas mice deficient in resistin are protected from obesity associated insulin resistance [15]. In this study, we also demonstrated that recombinant resistin protein and TNF-α reduced glucose uptake in human macrophages and atorvastatin reversed the abnormal glucose uptake induced by resistin and TNF-α. Resistin may represent a novel link between metabolic signals, inflammation, and atherosclerosis [16].

Norata et al. have reported that plasma resistin levels are increased in the presence of metabolic syndrome and are associated with increased cardiovascular risk [17]. Lubos et al. have also reported that resistin levels are elevated in patients with acute coronary syndrome and might play a role as a diagnostic marker [18]. Recently, resistin was found to induce lipolysis and re-esterification of triacylglycerol stores and increase cholesteryl ester deposition in human macrophages [19]. Therefore, resistin contributes macrophage form cell formation. Statins have been shown to reduce lipid lowering effects as well as pleiotropic properties. Although statin cannot alter resistin levels in patients with type 2 diabetic and in healthy men [20-22], statins have been shown to reduce resistin expression in human monocytes and adipocytes [10,23]. These data implicate that statins may control inflammatory responses by inhibiting resistin expression.

Indeed, our study demonstrated that TNF-α induced resistin protein and mRNA expression in human macrophages and atorvastatin decreased TNF-α-induced resistin expression in a dose-dependent manner. The induction of resistin protein by TNF-α was largely mediated by JNK kinase pathway because the specific and potent inhibitors of an upstream JNK kinase, SP600125, inhibited the induction of resistin protein. Atorvastatin also inhibited the phosphorylation of rac induced by TNF-α. In this study, we demonstrated that TNF-α stimulation of AP-1-DNA binding activity required at least phosphorylation of the JNK since JNK inhibitor abolished the AP-1 binding activity. Atorvastatin also attenuated the AP-1 binding activity induced by TNF-α. The promoter activity of wild resistin promoter after TNF-α was significantly higher than that of AP-1 mutant resistin promoter. This finding indicates that TNF-α regulates resistin in human macrophages at transcriptional level and that AP-1 binding sites in the resistin promoter is essential for the transcriptional regulation. Taken together, our results indicate that TNF-α may increase the AP-1 transcriptional activity in macrophages. Resistin induced by TNF-α was largely though JNK, rac and resistin promoter pathways and atorvastatin could inhibit resistin expression through inhibition of rac phosphorylation, reduced AP-1 binding activity and resistin promoter activity.

In our study, we demonstrated that inhibition of the TNF-α-induced resistin expression by atorvastatin was independent of HMG-CoA reductase. Downregulation of resistin expression induced by CRP by simvastatin was independent of HMG-CoA reductase [23]. Rac pathway mediates the signal trasndcution by isoprenoid intermediates, such as farnesylpyrophosphate and geranylgeranyl-pyrophosphate. In this study, we did not test the effect of isoprenoid intermediate on inhibition of TNF-α-induced resistin expression by atorvastatin. We demonstrated that TNF-α and mevalonate induce resisitn at the similar level. However, atorvastatin only blocks TNF-α but not mevalonate induced resistin. TNF-α but not mevalonate induces rac phosphorylation in cultured macrophages. JNK specific inhibitor S600125 blocked both TNF-α and mevalonate induced resistin expression. This data suggests that mevalonate plays a proinflammatory role and atorvastatin attenuates resistin expression induced by TNF-α is independent of HMG-CoA reductase but through inhibition of Rac and JNK pathway.

In conclusion, our study confirmed previous findings that TNF-α could induce resistin expression in human macrophages and atorvastatin could inhibit the resistin expression induced by TNF-α. The inhibitory effect of atorvastatin on TNF-α-induced resistin expression was mediated by rac and resistin promoter. Our findings provide another evidence of pleiotropic effect of statin. Statin therapy may become another therapeutic strategy for controlling resistin-associated pathologic cardiovascular disease in humans.

## Abbreviations

AP-1: activating protein 1; EMSA: electrophoretic mobility shift assay; HMG-CoA reductase: 3-hydroxy 3-methyl glutaryl-CoA reductase; PBMCs: peripheral mononuclear cells; TNF-α: tumor necrosis factor-α.

## Competing interests

The authors declare that they have no competing interests.

## Authors' contributions

KGS has participated in the design of the study and drafted the manuscript. SKC has made substantial contributions to conception and design, or acquisition of data, or analysis and interpretation of data. BWW has made substantial contributions to conception and design, or acquisition of data, or analysis and interpretation of data. PK has given final approval of the version to be published.

## Supplementary Material

Additional file 1 **Figure S1**. Expression of JNK, ERK and p38 MAP kinase in cultured macrophages. (A) Representative Western blot for phosphorylated and total JNK, ERK, and p38 MAP kinase in macrophages after treatment with TNF-α for various periods of time with or without inhibitor. (B) Quantitative analysis of phosphorylated protein levels. The values from treated macrophages have been normalized to matched GAPDH and corresponding total protein measurement and then expressed as a ratio of normalized values to each phosphorylated protein in control cells (n = 3 per group). **P < 0.001 vs. control. *P < 0.01 vs. control. ^‡^P < 0.001 vs. 6 hr. Click here for file

Additional file 2 **Figure S2**. Expression of Rac in cultured macrophages. (A) Representative Western blot for phosphorylated and total Rac in macrophages after treatment with mevalonate for various periods of time. (B) Quantitative analysis of phosphorylated protein levels. The values from treated macrophages have been normalized to matched GAPDH and corresponding total protein measurement and then expressed as a ratio of normalized values to each phosphorylated protein in control cells (n = 3 per group). Click here for file

Additional file 3 **Figure S3**. Effect of recombinant resistin and TNF-α on glucose uptake in macrophages. Glucose uptake was measured in macrophages treated for 90 min with 20 μg/mL recombinant mouse resistin or 1 ng/mL TNF-α with or without resistin siRNA or atorvastatin. *P < 0.001. Data are from 3 independent experiments. Click here for file
