# Supplementary material for: Mechanism of inhibitory effect of atorvastatin on resistin expression induced by tumor necrosis factor-α in macrophages
Source: J Biomed Sci. 2009 May 27;16(1):50. doi: 10.1186/1423-0127-16-50 (PMC2694160; doi:10.1186/1423-0127-16-50)
Supplement: Additional file 1 — Figure S1. Expression of JNK, ERK and p38 MAP kinase in cultured macrophages. (A) Representative Western blot for phosphorylated and total JNK, ERK, and p38 MAP kinase in macrophages after treatment with TNF-α for various periods of time with or without inhibitor. (B) Quantitative analysis of phosphorylated protein levels. The values from treated macrophages have been normalized to matched GAPDH and corresponding total protein measurement and then expressed as a ratio of normalized values to each phosphorylated protein in control cells (n = 3 per group). **P < 0.001 vs. control. *P < 0.01 vs. control. ‡P < 0.001 vs. 6 hr. [file 1423-0127-16-50-S1.ppt]

## Slide 1
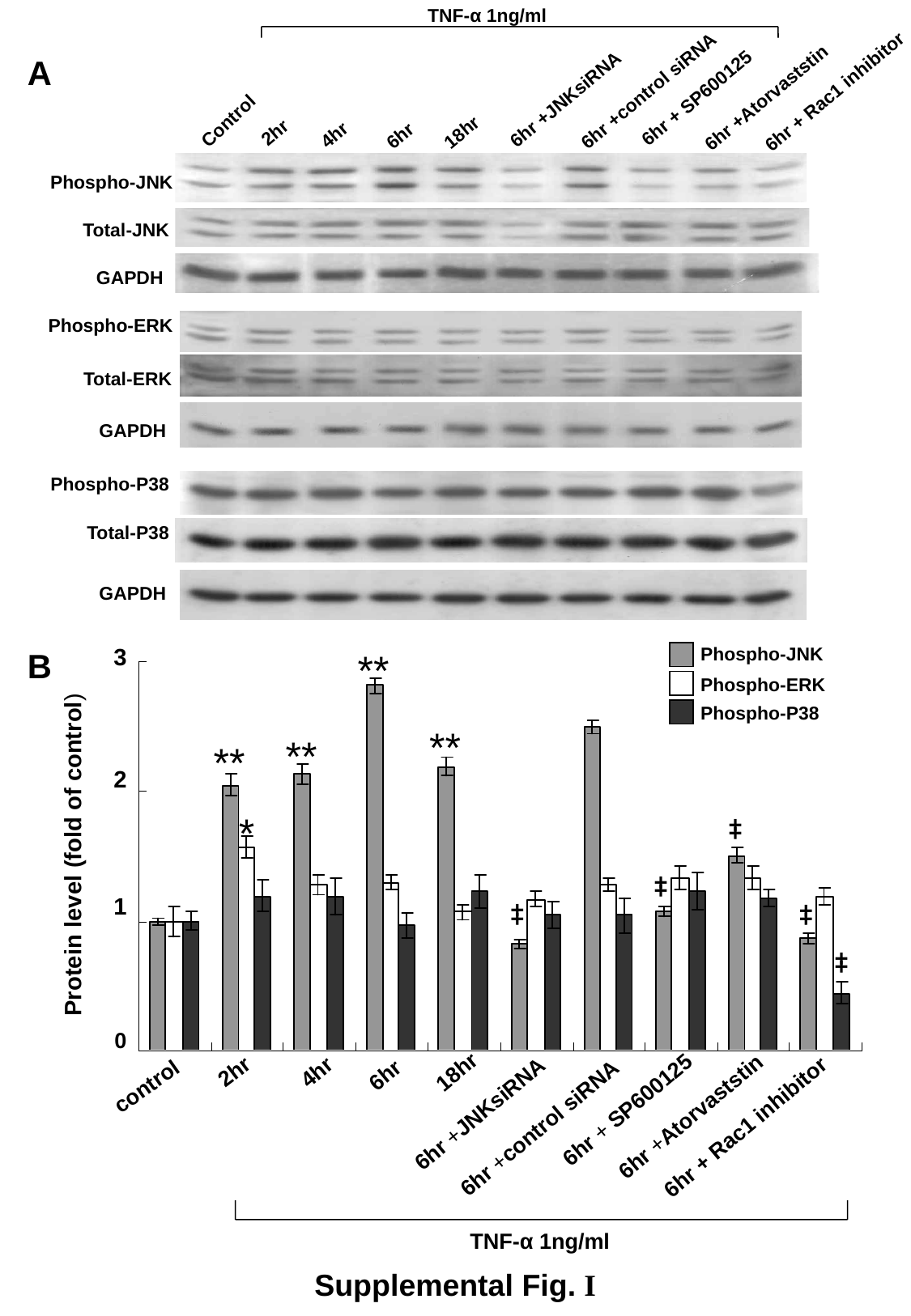

TNF-α 1ng/ml
6hr +control siRNA
A
6hr + Rac1 inhibitor
6hr +JNKsiRNA
6hr + SP600125
6hr +Atorvaststin
Control
4hr
2hr
6hr
18hr
Phospho-JNK
Total-JNK
GAPDH
Phospho-ERK
Total-ERK
GAPDH
Phospho-P38
Total-P38
GAPDH
3
Phospho-JNK
Phospho-ERK
Phospho-P38
B
**
**
**
**
2
*
+
+
Protein level (fold of control)
+
+
1
+
+
+
+
+
+
0
2hr
18hr
6hr
4hr
control
6hr + SP600125
6hr +Atorvaststin
6hr +JNKsiRNA
6hr + Rac1 inhibitor
6hr +control siRNA
TNF-α 1ng/ml
Supplemental Fig. I
