# Supplementary material for: Mechanism of inhibitory effect of atorvastatin on resistin expression induced by tumor necrosis factor-α in macrophages
Source: J Biomed Sci. 2009 May 27;16(1):50. doi: 10.1186/1423-0127-16-50 (PMC2694160; doi:10.1186/1423-0127-16-50)
Supplement: Additional file 2 — Figure S2. Expression of Rac in cultured macrophages. (A) Representative Western blot for phosphorylated and total Rac in macrophages after treatment with mevalonate for various periods of time. (B) Quantitative analysis of phosphorylated protein levels. The values from treated macrophages have been normalized to matched GAPDH and corresponding total protein measurement and then expressed as a ratio of normalized values to each phosphorylated protein in control cells (n = 3 per group). [file 1423-0127-16-50-S2.ppt]

## Slide 1
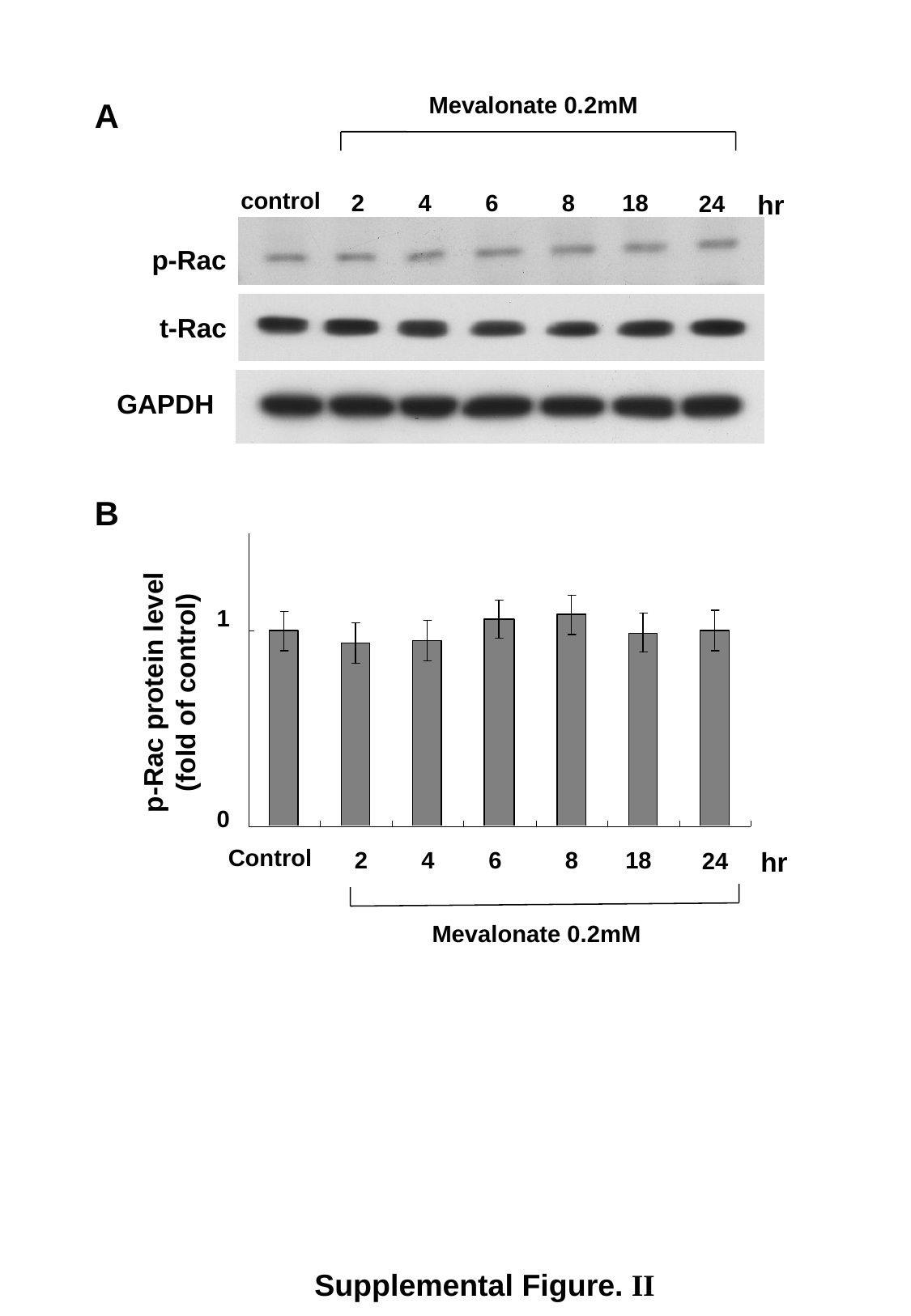

Mevalonate 0.2mM
A
control
2
4
6
8
18
hr
24
p-Rac
t-Rac
GAPDH
B
1
p-Rac protein level
(fold of control)
0
Control
2
4
6
8
18
hr
24
Mevalonate 0.2mM
Supplemental Figure. II
