# Supplementary figures and images for: Mechanism of inhibitory effect of atorvastatin on resistin expression induced by tumor necrosis factor-α in macrophages
Source: J Biomed Sci. 2009 May 27;16(1):50. doi: 10.1186/1423-0127-16-50 (PMC2694160; doi:10.1186/1423-0127-16-50)

## Slide 1
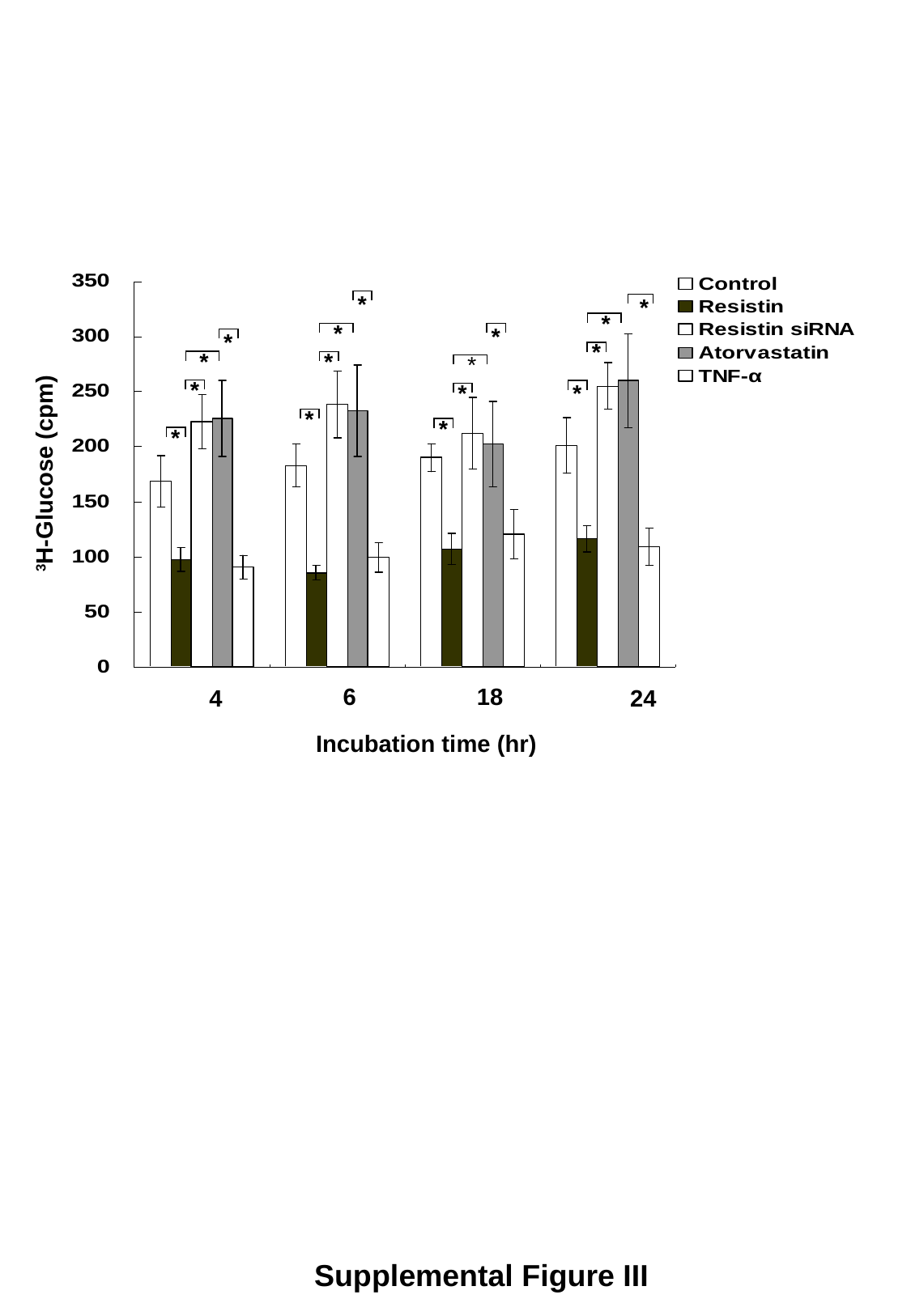

*
*
*
*
*
*
*
*
*
*
*
*
*
*
*
*
 3H-Glucose (cpm)
6
18
4
24
Incubation time (hr)
Supplemental Figure III

Supplement: Additional file 3 — Figure S3. Effect of recombinant resistin and TNF-α on glucose uptake in macrophages. Glucose uptake was measured in macrophages treated for 90 min with 20 μg/mL recombinant mouse resistin or 1 ng/mL TNF-α with or without resistin siRNA or atorvastatin. *P < 0.001. Data are from 3 independent experiments. [file 1423-0127-16-50-S3.ppt]
